# EHMTI-0081. Use of rotigotine in chronic cluster headache

**DOI:** 10.1186/1129-2377-15-S1-C12

**Published:** 2014-09-18

**Authors:** L Savi, C Condello, L Pinessi

**Affiliations:** 1 Headache Center, Città della salute e della scienza, Torino, Italy

## Background

Chronic Cluster Headache (CCH) is an infrequent form of Cluster Headache, defined by the absence of remission periods. Medical treatments are few, sometimes ineffective and poorly tolerated. Rotigotine was recently reported as useful in a single patient with CCH.

## Aim

Reporting the results observed using rotigotine in four cases with CCH.

## Cases report

All patients received a diagnosis of CCH according to ICHD-3 Beta. Brain imaging and neurological examination were normal. Patients were 61, 67, 49 and 45 respectively. Verapamil, carbolithium and steroids were used unsuccessfully.

Case 1: he had tried Gammacore and pregabalin, too, ineffectively. Transdermal rotigotine was started at the dose of 2 mg/die. After a few days, only scarce, minor attacks persisted, and were stopped by titrating posology to 4 mg/die.

Case 2: rotigotine was titrated up to 6 mg/die, without any beneficial effect, and after a month it was discontinuated.

Case 3 and 4: titration proceded to 4 mg/die, for persistence of minor attacks with lower doses. Lasting remission has been observed since.

No adverse event has been reported by the patients.

## Conclusions

Rotigotine is a non-ergoline D3-like receptor agonist. Its availability in the transdermal form and its safety profile make it well accepted by patients. The four subjects whose cases we reported did not show any adverse event. Three of them showed initial but definite benefit from this therapy and are currently free from pain and under follow-up. We think that rotigotine should be considered in the management of CCH unresponsive to common treatment.

No conflict of interest.

